# Improving Middle-Income Countries Access to Vaccines. A Blueprint to Overcome Current Challenges

**DOI:** 10.5334/aogh.4151

**Published:** 2023-11-22

**Authors:** Carlos Espinal, Francisco Becerra-Posada, Jaime R. Torres

**Affiliations:** 1 Global Health Consortium (GHC), Department of Global Health - Robert Stempel College of Public Health & Social Work - Florida International University, US; 2 Infectious Diseases Section, Tropical Medicine Institute, Universidad Central de Venezuela, Venezuela

**Keywords:** Vaccine access, vaccine research, vaccine innovation, research and development

## Abstract

The Global Health Consortium at Florida International University developed an end-to-end solution framework based on the input of a diverse panel of experts from middle-income country (MIC) government agencies, public health think tanks, academia, and nonprofit organizations to identify mechanisms to help MIC governments and stakeholders increase access to novel vaccines for infectious diseases. The resultant layout can be deployed to improve vaccine discovery and development, strengthen regulatory processes, and boost vaccine production, access, and implementation. Mechanisms include policies and incentives MIC governments can use to stimulate vaccine investment and activity, as well as actions government agencies can take together with other stakeholders to coordinate efforts or build capabilities. Through a series of individual virtual interviews, we engaged diverse experts from MIC government agencies, public health think tanks, academia, and nonprofit organizations who understand the vaccine ecosystem, immunization policies, and population health financing at global, regional, and country levels. Responses were mapped, and in-depth questions were prepared for a group virtual discussion. This paper is the result of such a group discussion. The panel identified clear opportunities for MICs to improve locally-driven innovations and future access to novel vaccines. It proposes a solution framework for countries considering investing in vaccine research and development and innovation to use as a guide to evaluate the steps they could take to improve such an environment and incentivize innovation in vaccine development. It is hoped that this end-to-end solution framework will become a key resource to help MICs strengthen policies and take more actions to make such improvements.

## Introduction

Recent available data from WHO and UNICEF based on estimates of national immunization coverage indicate that more than one out of four children in middle-income countries (MICs) fails to complete the basic vaccine schedule. As a result, about 25 million children, more than 60 percent of them living in just 10 countries (India, Nigeria, Indonesia, Ethiopia, the Philippines, the Democratic Republic of the Congo, Brazil, Pakistan, Angola, and Myanmar), were un- or under-vaccinated in 2021. Worse still, in the same year, more than 18 million children did not receive any vaccines (zero-dose children), an increase of 5 million from 2019, a situation worsened by the pandemic when many children were not properly vaccinated [1].

As new vaccines become available, more countries are making efforts to incorporate them into their immunization programs. However, issues such as unaffordability, lack of technical capacity in forecasting and planning, unreliability of local vaccine suppliers, and limited and/or unpredictable funding represent serious challenges for MICs to achieve universal immunization coverage.

The emergence of the COVID-19 pandemic represented an unprecedented global challenge with appalling health and socioeconomic consequences. The rapid development and production of effective vaccines against the disease over the past two years offered a unique opportunity to reduce its impact; however, vaccines did not reach all parts of the world equally, curtailing the possibility of effectively controlling viral spread. Indeed, according to the information collected by the WHO and the United Nations, most vaccine doses were distributed in high- and upper-middle-income countries, while other most-needed areas, especially African countries, still lag in the vaccination process [2, 3].

Lack of vaccination due to unequal access generates economic and social disadvantages, which could instead increase other inequalities [4]. In this regard, COVID-19 vaccine inequality will continue to profoundly impact socio-economic recovery in low-income countries if urgent actions to assure equitable access worldwide are not taken; it would also delay progress towards the Sustainable Development Goals (SDGs) [5,6,7,8].

Many activities are underway to increase equitable access to COVID-19 vaccines and improve preparations for future pandemics. Global actors and institutions are driving new collaborations, policies, and funding mechanisms to deliver COVID-19 vaccines and increase support for low-income countries (LICs) and MICs. In parallel, efforts at country and regional levels are exploring ideas to improve vaccine procurement, manufacturing capacity, and implementation. This presents an opportunity to help MICs look beyond pandemics to strengthen policies and actions that drive vaccine innovation and access for unsolved infectious diseases affecting developing countries.

## Methodology

During the first half of 2022, the Florida International University Global Health Consortium organized the Access to Vaccine Innovation Framework (AVIF), engaging diverse experts with a background in vaccine-related work, development, or regulatory frameworks from MIC government agencies, public health think tanks, academia, and nonprofit organizations who understand the vaccine ecosystem, immunization policies, and population health financing at global, regional, and country levels. A one-hour virtual interview was conducted with each of the 14 experts, followed by a 2.5-hour virtual roundtable with all participants.

Initial discussions focused on the challenging task of stimulating the development of novel vaccines for neglected tropical diseases (NTDs) and unsolved infectious diseases that mainly affect developing countries. While scientific technology is available, investments in new vaccines to prevent such diseases are constrained by a low return on investment (ROI) and a lack of aligned end-to-end incentives for R&D, access, and implementation. However, experts’ discussions revealed that MIC interest in improving vaccine technologies and capabilities is broader than NTDs. Many MICs want the ability to develop novel vaccines for future pandemics and endemics, as well as to produce several vaccines for their Expanded Program on Immunization/National Immunization Program (EPI/NIP).

The objective was to develop an end-to-end solution framework containing proposals for designing and implementing or adequations to policies and actions to help MIC governments, financing agencies, researchers, and other stakeholders increase access to future novel vaccines for infectious diseases that disproportionately affect developing countries.

Meeting expected outputs included a summary and discussion of:

**Barriers** – Identification of barriers that limit MIC stakeholders from doing more to encourage vaccine innovation and access

**Solution framework** – An end-to-end framework of policies, incentives, and actions across the continuum of vaccine discovery, development, sustainable access, and implementation that MIC stakeholders can utilize to advance capabilities and increase access to future novel vaccines

**Case for action** – Factors that motivate MIC governments and stakeholders to move forward on investments and improvements in locally driven innovations

In addition, the experts agreed on recommendations and important considerations to guide the utilization of this framework by MIC stakeholders based on country circumstances and goals.

The discussions and recommendations by the experts are grouped under I. Barriers impacting MIC Stakeholders, where several barriers were detected, and II. an End-to-End Solution Framework, structured into three sections to reflect the full continuum from vaccine discovery and development through sustainable access and implementation: a) Front-End Drivers of R&D Capabilities, b) Regulatory Drivers, and c) Downstream Market Drivers.

## Results

### Barriers Impacting MIC Stakeholders

Discussions among panel experts explored obstacles that limit MIC stakeholders from doing more to encourage vaccine innovation and access. This revealed multiple all-encompassing, front-end, regulatory, and downstream barriers throughout the MIC vaccine ecosystem, such as financing for R&D and economic constraints, political motivations and focus on the short term, stakeholder interests, over-reliance on multinational pharmaceutical corporations, regulatory weaknesses, and market size. Considerations are to be taken to overcome these barriers. (See Tables 1a–1b, 2).

**Table 1a:** All-embracing barriers and specific issues constraining vaccine development in low-and middle-income countries (LMCs).


ALL-EMBRACING BARRIER	SPECIFIC ISSUES

**Financial and economic constraints**	Economic restrictions worsened by COVID-19 pandemic Insufficient funding for scientific research and development (R&D) Low advocacy for vaccines budget

**Political focus is short term**	Politicians tend to favor near-term results, but investments in vaccine capabilities take years to bear fruit Easier to pursue near-term fill and finish manufacturing capabilities than investing in a long-term vaccine R&D strategy Changes in administrations and governments can cause shifting focus and funding

**Over-reliance on Multinational Corporations (MNCs) and global actors**	Historical reliance on MNCs and global actors as drivers of vaccine innovation was further accentuated during the COVID-19 pandemic Partnerships with MNCs are not balanced enough for MICs MNCs are not as focused on developing vaccines for NTDs

**Competition from other health priorities**	Vaccines compete against other health-related topics for limited funds and resources


**Table 1b:** Front-end and downstream barriers and specific issues constraining vaccine development in low-and middle-income countries (LMCs).


FRONT-END BARRIER	SPECIFIC ISSUES	*DOWNSTREAM BARRIER*	*SPECIFIC ISSUES*

**Undertaking of an R&D approach for vaccine development by a MIC may not be justified based on its expected commercial benefit**	• A novel vaccine must pay for itself Belief that it is cheaper to buy a vaccine than to develop one	***MICs single country markets are small; therefore, sustaining local production requires access to other countries markets***	*MIC production will need demand and volume from multiple countries* *It will take time for new MIC output to achieve competitive scale and pricing, leading to higher prices in the short term which may constrain access to export markets* *Technology transfer agreements are often bound to one country, disallowing exportation*

**Gaps in R&D and scientific capabilities**	Limited know-how for basic science Academic structure needs to be modernized Training is needed for researchers	***Focus on low price by pooled procurement agencies will constrain new vaccines developed by MICs***	*Pooled procurement agencies may limit exportation opportunities for new vaccines from MICs because of higher prices*

**Perception bias that MICs do not have sufficient R&D capabilities**	MICs are not seen as a reliable partner for conducting R&D MICs are mainly engaged by MNCs when there is a specific need (e.g., local clinical trial)	***Lack of capabilities and capacity for sustainable post-clinical trial production***	*It takes significant time to develop infrastructure, build and train a workforce, license technologies, and secure government contracts*

**Favorable intellectual property (IP) environment is not supporting or driving more innovation**	MICs with good IP laws are not seeing increases in technology transfer agreements or approval of new products	***Vaccine implementation challenges***	*Health system difficulties in turning available supply into application in target populations* *Training healthcare workers for vaccine implementation* *Concerns about increasing the complexity of the immunization schedule by adding more vaccines to the EPI/NIP* *Vaccine distrust and hesitancy hinders routine immunization rates and uptake of novel vaccines*


**Table 2:** Regulatory barriers limiting MICs stakeholders from achieving better results to foster vaccine innovation and access.


REGULATORY BARRIER	SPECIFIC ISSUES

**Regulatory limitations delay vaccine approval and access for MICs**	Lack of stringent regulatory agencies in the Global South*, affects timing to review and approve vaccines, which vaccines countries have access to, availability of vaccines for exportation, and impacts the willingness of stakeholders to invest in R&D. In Latin America at least six countries (Argentina, Brazil, Chile, Colombia, Cuba and Mexico) with level 4 regulatory agencies, could be further strengthened to become a stringent supervisory body for the region *Pooled procurement agencies^ may paradoxically limit exportation opportunities for new vaccines from MICs because of regulatory aspects and WHO prequalification process*


Assessing which barriers any particular MIC is facing is an important step to fine-tune the application of the end-to-end solution framework in order to aim for improvement in specific capabilities.

### End-to-end Solution Framework to Help MICs Increase Access to Future Novel Vaccines

During the AVIF interviews and roundtable, participants in the expert panel identified numerous mechanisms that have successfully driven vaccine innovation, access, and implementation. Input was based on direct experiences working in LMICs, knowledge of what works well in benchmark high-income countries, as well as lessons learned throughout the COVID-19 pandemic. The resulting current end-to-end solution framework is structured into three sections that reflect the full continuum from vaccine discovery and development through sustainable access and implementation.

Each section was further organized into specific objectives, underneath which are corresponding mechanisms to help MICs strengthen their capabilities to advance vaccine innovation. Each objective involves two types of mechanisms: policies and incentives for an MIC government to encourage actions or investments by other stakeholders, and recommended actions that MIC government agencies, together with other stakeholders, can implement to coordinate efforts or build or expand capabilities. Overall, four objectives were presented for discussion under three main areas: Front-End Drivers for R&D Capabilities, Regulatory Drivers, and Downstream Market Drivers (Table 3).

**Table 3:** End-to-end Solution Framework to Help MICs Increase Access to Future Novel Vaccines.


CATEGORIES	FRONT END DRIVERS OF R&D CAPABILITIES	REGULATORY DRIVERS	DOWNSTREAM MARKET DRIVERS

Objectives	1: Create a long-term government strategy & vision for access to vaccine innovation 2: Strengthen R&D know-how and capabilities throughout a MIC 3: Collaborate for specific infectious diseases 4: Spur vaccine R&D through investment, funding, and other incentives/mechanisms	1: Strengthen regulatory processes in and across MICs 2: Create regulatory incentives to accelerate vaccine innovation	1: Create incentives through procurement commitments 2: Leverage regional pooled models for innovation 3: Create incentives through tiered pricing 4: Support sustainable access and vaccine implementation


For the Front-End Drivers for RD Capabilities, four objectives were suggested for discussion for recollecting recommendations on actions and/or policies and incentives by addressing the suggested objectives (Table 3). Among the suggested actions for governments were to define a long-term State vision for vaccine R&D and production, place focus on improving weaker areas of the vaccine chain, increase investment and funding for vaccine R&D platforms and research centers, and exercise a coordinating role and research priority setting among all stakeholders.

Policies and incentives are to be strengthened by fostering the formation of future scientists and researchers, enabling the incorporation of researchers from other countries, incentivizing technology transfers, and tying fund allocation to universities and research centers to their research efforts. Regarding specific actions recommended to be implemented, these were to forge R&D partnerships and consortia with key stakeholders, establish centers to train a workforce for vaccine R&D and production, such as the Partnerships for African Vaccine Manufacturing (PAVM), conduct science and vaccine diplomacy to link global actors, and participate in learning and best practice networks.

The need to explore and form Public-Private Partnerships (PPP), and/or Production Development Partnerships to increase capabilities and encourage partnerships to accelerate vaccine development, mainly with academic institutions working around vaccines, was stressed.

Experts recommended increasing government funding for vaccine R&D through various mechanisms, providing tax incentives, tax credits or subsidies to those developing vaccines or investing in vaccine R&D, as well as de-risking through multiple funding sources, such as the Right Fund in Korea, pooling regional funds for R&D and innovation, risk sharing with high-income countries and companies, and embedding vaccine experts in governments, parliamentary roles, and diplomacy.

Regarding the Regulatory Drivers, a much-needed perception, not only in countries but multi-country, is that strengthening regulatory agencies towards a fast WHO prequalification is essential, as is improving regional regulatory capabilities and harmonization. The lack of a regional regulatory body, such as EMA, weakens the regions; however, Africa has taken steps towards implementing the new African Medicines Agency, and Mexico has proposed, during the recent 7th Summit of Heads of State and Government of the Community of Latin American and Caribbean States (Celac), the creation of a Latin American Medicines Agency [9, 10].

Countries should cooperate in facilitating a practical network to share advances and common knowledge to foster regulatory processes both at country and regional levels. They also should aim to apply policies that incentivize the acceleration of vaccine innovation by rewarding successful producers by various methods and providing an exclusive extended period to limit competition and reward local developers. An expedited review, such as the US FDA Fast Track review, could be implemented.

Another aspect to consider is the need to have strong intellectual property laws along with proper management and enforcement for significant R&D investments and to protect scientific innovations.

When discussing the Downstream of Market Drivers, focus was placed on promoting incentives through advance market commitments to procure a certain volume of a vaccine for an established price once it meets certain conditions. Higher purchase commitments are needed as an incentive for more involved local production that goes beyond the fill-and-finish stage.

Pooled funding through a special program to create incentives for multi-national corporations to develop vaccines for NTDs and secure a return on investment ought to be explored, as well as leveraging regional pooled models of innovation by creating a regional pooled procurement capability that encourages MIC vaccine innovation and exportation through new models incentivizing the development of novel vaccines [11].

MICs countries may also benefit from the pooling of emergency response funds to stockpile vaccines and investment in solutions for future pandemics that could be started immediately, as ASEAN countries are already discussing.

Tier pricing is seen as an effective mechanism that provides incentives for vaccine producers and affordability for countries based on differing income levels. An attractive and sustainable trade environment is needed by reducing restrictions, import duties, and taxes on vaccine inputs.

A key element is the support of sustainable access and vaccine implementation by national health systems and services, along with training to overcome vaccine implementation barriers. Countries should strengthen or incorporate laws to protect funding for new vaccines in their current schedules, secure financing for novel vaccines, and improve vaccine coverage. Plans should be set in place to secure the introduction of emergency vaccines and the implementation of novel vaccines, considering technical training as well as a communication strategy. Lessons learned should be shared through community practice.

#### Case for action

In addition to providing input on the policies and actions in the solution framework, program participants identified three primary motivations that compel MICs governments and stakeholders to take actions that improve access to vaccine innovations. (Table 4) Countries will need to have strong data and perform the needed analysis to move forward, making sure they can improve the vaccine landscape locally and/or regionally. To support this, WHO recently developed the Full Value of Vaccine Assessments (FVVA) framework to help country authorities evaluate vaccine value beyond individual health benefits and include the broader socioeconomic and indirect impacts mentioned above, along with strong economic modeling [12].

**Table 4:** Case for action.


CATEGORIES	INFECTIOUS DISEASES ARE SEEN AS A PRIORITY BY THE GOVERNMENT	VACCINE SELF-RELIANCE AS AN ESSENTIAL ENABLER OF HEALTH AND ECONOMIC SECURITY	VACCINES AND BIOTECHNOLOGY ARE STRATEGIC PRIORITIES FOR COUNTRY OR REGIONAL ECONOMIC GROWTH

**Relevant comments**	Government stakeholders must be convinced that the burden of an infectious disease makes preventing it a high priority. Decision makers must have access to data and projections to understand the full impact and value of prevention. This requires a strong evidence-based business case and health economic modeling to support MIC government decision making.	Throughout the pandemic, MICs were reliant on MNCs, NGOs, and HICs for COVID-19 vaccines and had to either wait their turn or fend for themselves to receive supply. MICs want the ability to develop novel vaccines for future pandemics/endemics, and to produce several vaccines on their EPI/NIP. This is motivating MICs to make investments and deepen partnerships to improve their end-to-end vaccine value chain capabilities.	Some MIC governments view science, biotechnology, and vaccines as strategic enablers of economic growth. These MICs want to diversify their economic output beyond their historical base and shift toward a more knowledge-based economy.


There are important macro considerations to take into account, such as ensuring MICs long-term commitments and sustainable funding, like Panama and South Africa are doing. Regional banks could and should play a role in looking into sustainable funding, along with interested governments. Countries and regions should take advantage of the growth in interest and momentum in regional models for vaccine R&D and production. Along with this comes the strengthening of regulatory processes and requirements. Regional bodies, such as the regional offices of WHO, should play a key role.

Best practices and learning networks should be joined or developed. Knowledge sharing is key for innovation and scaling up vaccine R&D and production. Researchers’ exchange through extended visits, sabbaticals, or long contracts is key for development and innovation.

## Discussion

The presented set of recommendations is a proposed blueprint for countries to move ahead and foster vaccine innovation, R&D, and production. Experts understand that policies take time to be implemented and that actions have to be discussed and adapted to local contexts; however, now is the right time to start discussing and hopefully doing so with a vision of the State towards the long term and not as a governmental plan that might be changed. Political will is crucial to bring all stakeholders to the table, learn from those countries that have advanced on this path, and start implementing changes. This support is seen in how some countries have a clear path forward while others are not even considering vaccine production.

The end-to-end solution design proposed here should be engineered and evaluated to identify other potential specific barriers and the policies and actions that are most likely to work in each context, as implementation strategies are likely to differ across countries and regions.

The proposed framework should serve to: a) help MICs strengthen their environments for vaccine R&D; and b) further discuss this and other areas of opportunity to bring all interested stakeholders to the table and have meaningful discussions.

This framework contains mechanisms and actions that MICs can take advantage of to drive improvements spanning from vaccine discovery and development through vaccine implementation. Packaging these mechanisms into a holistic framework gives MICs an opportunity to apply them to targeted areas along the vaccine value chain. Which specific policies and actions an MIC chooses to deploy depends on an assessment of several factors. As presented here, approval of vaccines and incorporation into the national vaccination schedule will vary since each country’s sovereignty in decision-making will prevail.

Finally, a draft report is being finalized and will be used to further gather ideas from MICs related to vaccine R&D and validate the usefulness of the proposals. This paper aims to broaden the outreach of the proposal and incite discussion.
